# Multi-Level Resistive Switching in SnSe/SrTiO_3_ Heterostructure Based Memristor Device

**DOI:** 10.3390/nano12132128

**Published:** 2022-06-21

**Authors:** Tsz-Lung Ho, Keda Ding, Nikolay Lyapunov, Chun-Hung Suen, Lok-Wing Wong, Jiong Zhao, Ming Yang, Xiaoyuan Zhou, Ji-Yan Dai

**Affiliations:** 1 Department of Applied Physics, The Hong Kong Polytechnic University, Hong Kong, China; tsz-lung-edward.ho@connect.polyu.hk (T.-L.H.); ke-da.ding@connect.polyu.hk (K.D.); nik.lyapunov@connect.polyu.hk (N.L.); chun-hung-dennis.suen@connect.polyu.hk (C.-H.S.); 19110062r@connect.polyu.hk (L.-W.W.); jiong.zhao@polyu.edu.hk (J.Z.); kevin.m.yang@polyu.edu.hk (M.Y.); 2 College of Physics, Chongqing University, Chongqing 401331, China; xiaoyuan2013@cqu.edu.cn

**Keywords:** memristor, RRAM, perovskite, SrTiO_3_, SnSe

## Abstract

Multilevel resistive switching in memristive devices is vital for applications in non-volatile memory and neuromorphic computing. In this study, we report on the multilevel resistive switching characteristics in SnSe/SrTiO_3_(STO) heterojunction-based memory devices with silver (Ag) and copper (Cu) top electrodes. The SnSe/STO-based memory devices present bipolar resistive switching (RS) with two orders of magnitude on/off ratio, which is reliable and stable. Moreover, multilevel state switching is achieved in the devices by sweeping voltage with current compliance to SET the device from high resistance state (HRS) to low resistance state (LRS) and RESET from LRS to HRS by voltage pulses without compliance current. With Ag and Cu top electrodes, respectively, eight and six levels of resistance switching were demonstrated in the SnSe/SrTiO_3_ heterostructures with a Pt bottom electrode. These results suggest that a SnSe/STO heterojunction-based memristor is promising for applications in neuromorphic computing as a synaptic device.

## 1. Introduction

With the development of the modern world, human society has entered a new era of big data and the Internet of Things (IoT), which brings great challenges and demands to computers for data processing [1,2]. As the demand for high-performance computers increases, the traditional Von Neumann architecture computer becomes less efficient since it separates the memory and calculation modules, leading to large and complicated hardware, high energy consumption, and long memory accessing time [3,4]. Resistive random-access memory (RRAM) is considered as one of the most desirable devices for emerging computer architecture due to its smaller size, faster response, higher endurance, non-volatile characteristics, and multilevel storage potential [5,6,7,8].

For RRAMs, they can be classified into two types by their working mechanisms: valence change mechanisms (VCM) triggered by the drift-diffusion of oxygen anions and electrochemical metallization mechanism (ECM) relying on the electrochemical dissolution and deposition of active electrode metals [9,10,11,12,13,14]. Both mechanisms play significant roles in resistive switching devices, and more studies are being carried out to achieve a deeper understanding of the mechanisms. Recently, it was found that, beyond those active metals, semiconductors can be used as a buffer layer between the metal electrode and the insulating layer in the memory devices, leading to interesting switching performance [3,4,5,8,15,16]. The performance of such complicated heterostructures may significantly change the memory characteristics such as operation voltage, resistance ratio, and multilevel storage performance etc., especially when choosing unconventional materials such as perovskite oxides SrTiO_3_ (STO) and BaTiO_3_ (BTO) as switching layers [17,18,19,20,21,22].

STO is a potential candidate for the next generation of high-*k* gate dielectric materials due to its large dielectric constant, low dielectric loss, and potential ferroelectricity under strain [23,24,25]. For RRAMs with STO thin films epitaxially grown on silicon, or a heterostructure of doped STO films on a Nb-doped STO substrate, their resistance on/off ratios are only in the range between 1 and 100 [18]. These low resistance ratios may be increased by stacking with a semiconducting buffer layer, forming a heterojunction structure. A heterostructure may also make the device achieve a much higher resistance ratio and even multilevel resistive switching.

In order to explore the switching mechanism and induce multilevel resistance states in the STO thin film-based device stacking with other semiconductor materials and different top electrodes, in this work, we report on SnSe/STO heterostructure-based memristor devices where the SnSe thin film is constructed in metal–insulator–metal (MIM) sandwich structures with Ag and Cu as top electrodes. Stoichiometric SnSe is a narrow band gap semiconductor at 0.9–1.3 eV, with relatively high resistance and a low-density of intrinsic defects [26]. These properties may make the SnSe memory device multifunctional through the integration of light sensitivity [27,28] and the ability to harvest energy from heat [29,30]. Our previous results show that SnSe is a good candidate for ultralow switching and multimode memristors [31]. In this work, for the first time, we demonstrate multilevel switching performance and resistive switching characteristics by inserting a SnSe film on a STO thin film to form a double layer heterostructure memristor device. We also studied the switching mechanism and compared the performance between using Ag and Cu as active electrodes. Furthermore, multilevel storage was achieved, and its outstanding performance as a memristor device has great potential in practical applications in neuromorphic computing (see the summarized performance comparison in Table 1).

## 2. Materials and Methods

**Figure 1:**
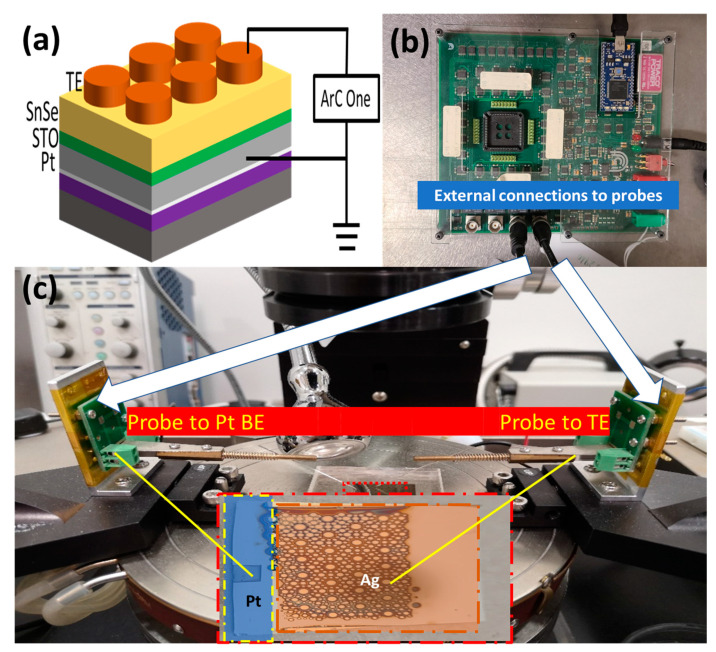
(**a**) Schematic of the TE/SnSe/STO/Pt memristor device with Cu or Ag top electrode (TE) connected to an ArC-One measurement instrument, (**b**) appearance of the ArC-One measurement instrument, and (**c**) the probe station and the measured devices.

First, a 30 nm thick STO film was deposited by laser molecular beam epitaxy (LMBE) at 625 °C on a 15 mm × 15 mm Pt/SiO_2_/Si (HF-Kejing, China) substrate. After reaching a base vacuum of 2 × 10^−5^ Pa, high-purity oxygen (O_2_: 99.99%) was flowed into the chamber during the deposition. For STO thin film deposition, an oxygen partial pressure of 5 Pa was maintained, which is an important factor for the property control. After finishing the deposition of STO film, the sample was cooled down from 625 °C to room temperature in 1000 Pa oxygen pressure. Subsequently, the base vacuum of 2 × 10^−5^ Pa was resumed, and a 90 nm thick SnSe film was in situ deposited at room temperature on the STO film to form a SnSe/STO heterostructure. In order to compare the efficiencies of the different electrodes, Ag and Cu top electrodes were selected and deposited on the SnSe film by direct current (DC) magnetron sputtering at room temperature through a shadow mask from 80 to 500 µm in diameter. The thicknesses of the Ag and Cu top electrodes were about 80 and 180 nm, respectively. Figure 1a shows the schematic diagram of the fabricated SnSe/STO heterostructure-based memristor for electrical characterizations performed at room temperature in air using an ArC-ONE memristor characterization instrument connected with a probe station. The thicknesses of the electrode layers were measured by means of a surface profiler (DektakXT, Bruker, Breman, Germany). The microstructure of the heterostructure was characterized by means of transmission electron microscope (TEM) (JEOL 2100F).

All of the first-principles calculations were performed using the Vienna Ab initio Simulation Package (VASP.5.4.4.18) with generalized gradient approximation of the Perdew–Burke–Ernzerhof functional (GGA-PBE) and projector augmented wave (PAW) potentials [35,36,37]. The DFT-D3 method was used in SnSe bulk to correct the van der Waals interactions [38]. The PBE+U method was applied in STO bulk to describe the strong correlation of electrons on the Ti 3*d* orbital (U = 4.2 eV) [39,40]. The cutoff energy was set to 400 eV and the first Brillouin zones of SnSe and STO were sampled by 2×6×6 and 6×6×6 Monkhorst–Pack grids, respectively [41]. The energy convergence criterion was set to $10^{-4}$ eV and force convergence criterion to 0.05 eV/Å. The optimized unit cells of SnSe (a = 11.491 Å, b = 4.177 Å, c = 4.503 Å) and STO (a = b = c = 3.936 Å) were adopted to construct supercells (1×2×2 and 2×2×2) to calculate the diffusion barrier of Ag and Cu atom using the nudged elastic band (NEB) method [42], respectively.

## 3. Result and Discussion

**Figure 2:**
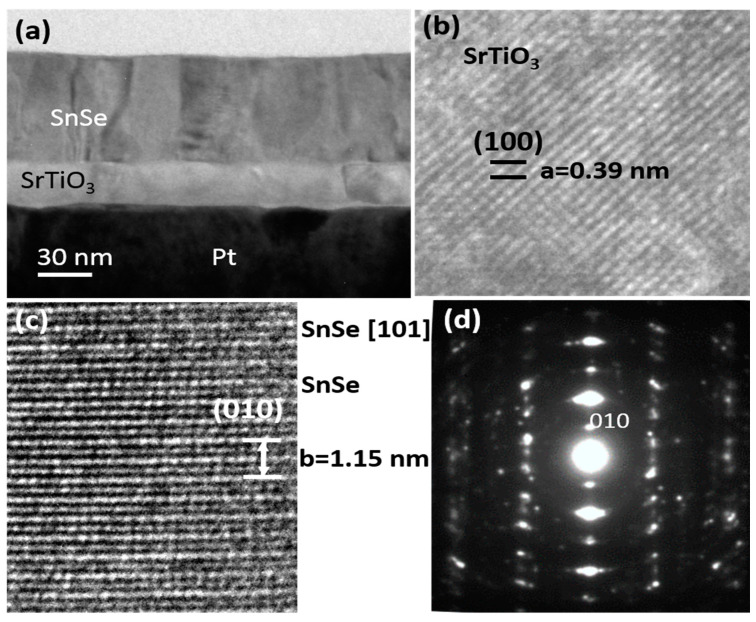
(**a**) A cross-sectional TEM image of a heterostructure device where the SnSe and STO layer can be clearly identified. (**b**) HRTEM image showing the crystalized structure of STO, HRTEM image (**c**), and diffraction pattern (**d**) of the SnSe layer viewed along the [101] direction.

The microstructure of the SnSe/STO/Pt heterostructure was characterized by a cross-sectional TEM study and the results are shown in Figure 2. The contrast in Figure 2a suggests that both the SnSe and STO layers are polycrystalline in microstructure, and the thicknesses of the SnSe and STO layers were determined to be about 90 and 30 nm, respectively. It is apparent that the SrTiO_3_ (STO) layer has a perovskite structure, as shown in Figure 2b, where the unit cell of STO can be identified and a lattice constant of a = 0.39 nm can be determined. Some areas of the polycrystalline SnSe layer presented a [10] preferred growth direction, and Figure 2c,d depict a typical HRTEM image and electron diffraction pattern along a zone axis close to the [101] direction. Electron dispersive x-ray (EDX) spectra (not shown) of both layers revealed nearly a 1:1 ratio of Sr:Ti and Sn:Se, suggesting stoichiometry of the deposited STO and SnSe layers.

**Figure 3:**
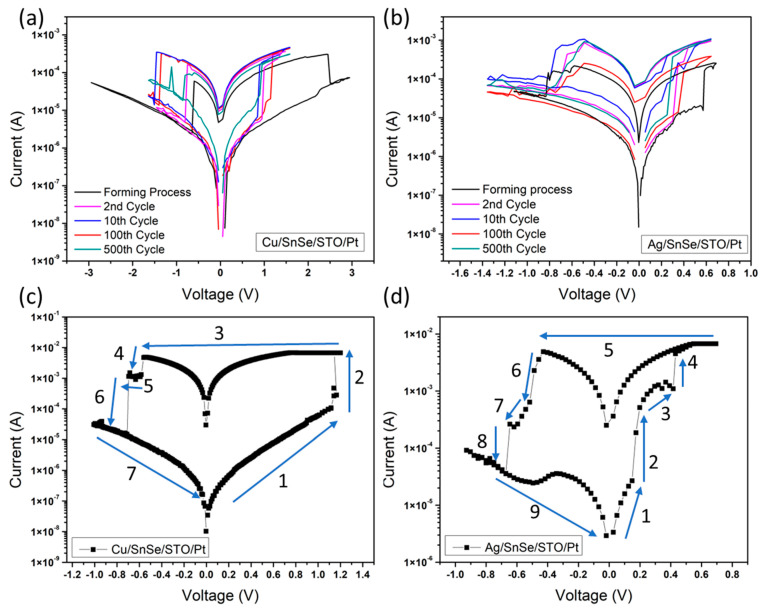
(**a**) The I–V curves of the Cu/SnSe/STO/Pt device and (**b**) the Ag/SnSe/STO/Pt device at the 500th applied voltage sweeps. The SET process and RESET process of the Cu/SnSe/STO/Pt device (**c**), and the Ag/SnSe/STO/Pt device (**d**).

The resistive switching characteristics of the Cu/SnSe/STO/Pt and Ag/SnSe/STO/Pt memory cells were investigated by voltage sweeping I–V curve measurements and the results are shown in Figure 3a,b. Generally, by steadily increasing the positive voltage imposed on the device from 0 V to a certain positive voltage, the device changes from a high resistance state (HRS) to a low resistance state (LRS) (i.e., the “SET” process). On the other hand, the “RESET” process occurs when the device changes from LRS to HRS by imposing the voltage from 0 V to negative voltage. As shown in Figure 3a, an electroforming process is required to activate the device to present the rectifying and hysteretic I–V characteristics, which is indicative of resistive switching. For both the Cu and Ag top electrode devices, a forming process is necessary to activate the memory effect. It should be pointed out that to protect the device from irreversible resistive switching or damage, we set a current compliance of 1.3 mA when applying the voltage sweeping on the device.

As shown in Figure 3a, by controlling the amplitude of the positive voltage sweep on the Cu electrode from 0 V→3 V →0 V with a 0.05 V step voltage increase for the forming process, we can see that the switching voltage was approximately at 2.5 V to SET the device from the initial state to LRS. After the forming process, the Cu/SnSe/STO/Pt device stays at the ON state. Then, a reset process of a negative voltage sweeps from 0 V→−3 V→0 V with a 0.05 V step voltage decrease was applied on the Cu electrode to switch the device back to the OFF state. The first RESET voltage was approximately −0.6 V to RESET the device from LRS to HRS. In order to study the reliability, we applied a voltage sweep from 0 V→1.6 V→0 V→−1.65 V→0 V with a 0.05 V step voltage for one thousand times. The 2nd, 10th, 100th, and 500th measured I–V curves of the Cu/SnSe/STO/Pt device showed that after five hundred times of switching, the on/off resistance ratio retained a high of about 10. The I–V characteristics of the Ag/SnSe/STO/Pt device measured by the same method are shown in Figure 3b. First, we applied a positive voltage sweep on the Ag electrode from 0 V→0.7 V→0 V with a 0.05 V step voltage for the forming process, where we could see that the device was switched ON at approximately 0.6 V. Then, a reset process of negative voltage sweeps from 0 V→−1.1 V→0 V with a 0.05 V step voltage was applied on the Ag electrode to switch the device back to the OFF state at approximately −0.8 V. We also repeated the switching process 1000 times on the Ag/SnSe/STO/Pt device to study the reliability. The results showed that after 500 times of switching, the on/off resistance ratio remained at about 10.

To analyze the switching mechanism, I–V curves of the switching process of both the Cu and Ag devices were measured, and the results are shown in Figure 3c,d. It is apparent that the switching process of the Cu/SnSe/STO/Pt device can be divided into seven steps. However, the switching process of the Ag/SnSe/STO/Pt device is much more complicated and contains nine steps. According to reference reports, an important aspect of metal oxide materials is the migration of oxygen vacancies (or oxygen ions) under an applied electrical field [9,10,11,12,22,43,44]. Based on the investigations from former studies, the switching mechanism in our devices should be mediated by the forming and rupture of the conductive filament. This active medium is actually much smaller than the cell size, which allows for a potential of scaling. When the positive voltage sweep is applied on both the Cu and Ag-electrode devices, oxidation occurs on the Ag and Cu top electrode since both are electrochemically active metals. Therefore, volatile Cu^+^ and Ag^+^ cations could form as Cu → Cu^2+^ + 2e^−^ and as Ag → Ag^+^ + e^−^. These volatile cations migrate toward the Pt electrode through the SnSe layer and STO layer and are finally reduced by electrons flowing from the cathode (i.e., Cu^2+^ + 2e^−^ → Cu and Ag^+^ + e^−^ → Ag. Meanwhile, when negative voltage sweeps were applied, Cu ions and Ag ions can migrate back to the top electrode in the same way.

However, because of the different electrochemical activities of Cu and Ag, the forming voltage of the Cu filament was relatively higher, and the switching time was relatively longer compared to the Ag filament formation [45,46]. The migration processes of the Cu filament and Ag filament in the SnSe layer and STO layer were different. In the Cu/SnSe/STO/Pt device, the 2nd step of the switching process showed that the Cu filament changed from the unstable state to stable state in the whole heterostructure. Before the Cu filament formed stably between the Cu top electrode and Pt bottom electrode, Cu ions first diffused into SnSe layer. After the Cu filament was stabilized in the SnSe layer between the Cu top electrode and the STO layer, it kept migrating to the Pt bottom electrode in the STO layer and finally changed the device to LRS. When the device was switched back from LRS to HRS in the 4th to 6th steps, the Cu filament ruptured and reduced back to the Cu top electrode. The filament first reduced in the STO layer and broke from the Pt bottom electrode, then it kept reducing in the STO layer, as shown in the 5th step. After the whole filament was reduced and broken in the STO layer, it started reducing at the SnSe layer and finally RESET to HRS, as shown in the 6th step. The switching process was very similar for the Ag/SnSe/STO/Pt device, but one could see that the 2nd to 4th steps, as shown in Figure 3d, presented a more complicated process when the Ag filament migrated between the SnSe layer and STO layer to switch the device to the ON state. Moreover, the RESET process of the Ag/SnSe/STO/Pt device in the 6th step showed a significant decrease in resistance, but the decreasing speed of resistance slowed down at the 7th step. This suggests that Ag ions migrate back from the STO layer to the SnSe layer and shows a multistate characteristic between the HRS and LRS. Finally, as the RESET voltage decreased, the Ag ions migrated back to the electrode and the device returned to the HRS. All of these switching processes show that these two devices may result in different resistance states, implying multilevel data storage and applications in neuromorphic computing.

**Figure 4:**
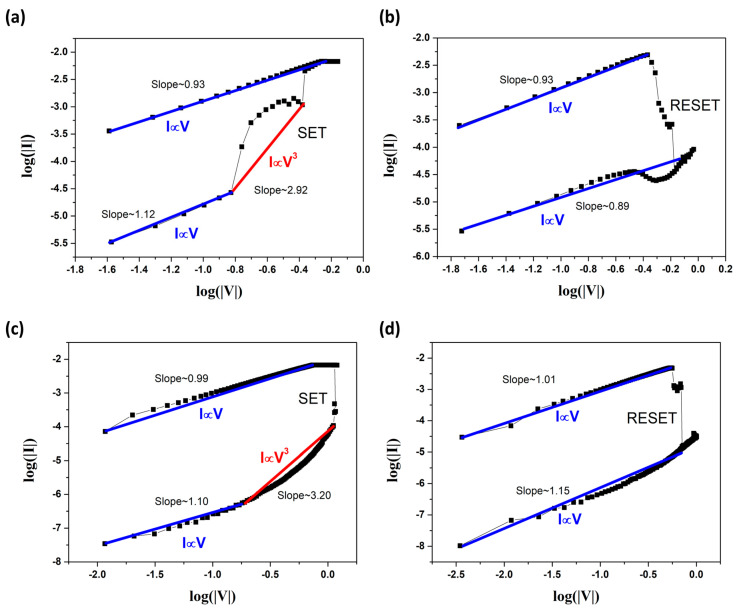
(**a**) Log(I)–Log(V) plots of the device with Ag TE under positive voltage sweeps and (**b**) under negative voltage sweeps. (**c**) Log(I)–Log(V) plots of the device with Cu TE under positive voltage sweeps and (**d**) under negative voltage sweeps.

We also created double logarithmic plots of I–V curves in both the positive voltage sweeps and negative voltage sweeps, as shown in Figure 4. One can see that during the SET process, as shown in Figure 4a,c, thermally generated carriers were dominant over the electrode-injected carriers in the ohmic conduction regime (I∝V) at low voltage. Then, the slope increased to the second regime (I∝V^3^) when the traps in SnSe/STO were all filled out by the carriers due to the further increase in the voltage. There were two distinctive regimes with different slopes in the SET process for both the Ag and Cu TE devices, which can be explained by the space charge limited conduction (SCLC) model. After the SET process, both devices were switched from HRS to LRS. As shown in Figure 4b,d, the double logarithmic plots of the I–V curves in the RESET process of both the Ag and Cu TE devices were also well-fitted with a slope of about 1, suggesting an ohmic conduction behavior. This good fit indicates that Ag ion-based bridge filaments and Cu ion-based bridge filaments are formed in the SnSe/STO heterostructure switching medium, where the current paths generated by filaments are metallic.

**Figure 5:**
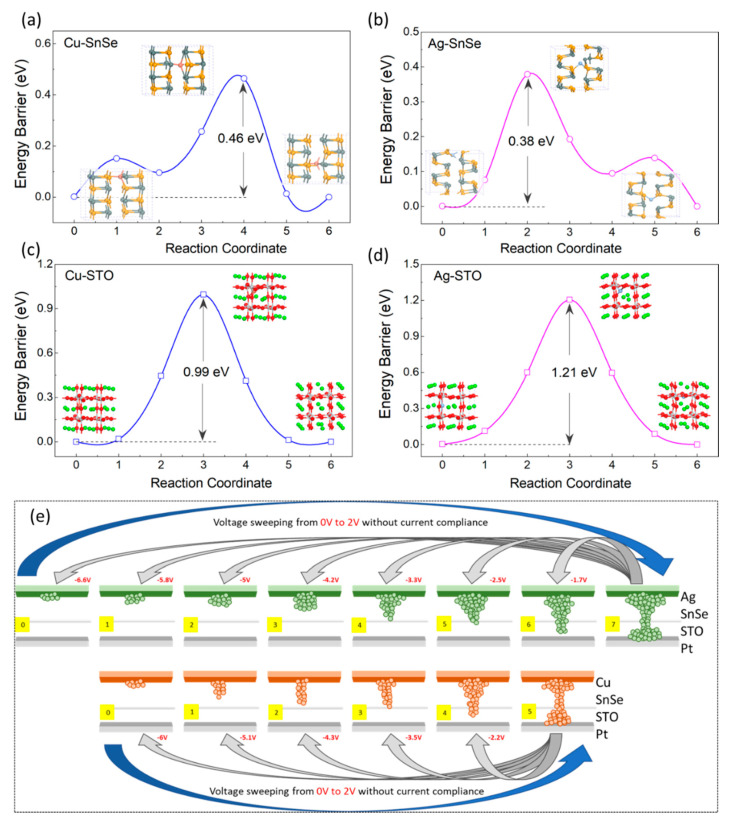
(**a**) The diffusion path and the energy barrier for Cu ions and (**b**) Ag ions diffused from the top electrode into SnSe. (**c**) The diffusion path and the energy barrier for Cu ions and (**d**) Ag ions diffused from the SnSe/STO interface into STO. (**e**) The switching mechanism of the Cu/SnSe/STO/Pt device and Ag/SnSe/STO/Pt device. The blue arrows indicate that both devices are switched from HRS to LRS by voltage sweeping without current compliance, and the gray arrow indicates that the multistate can be achieved by negative voltage pulse in different values.

To better understand the diffusion path and the energy barrier for Cu and Ag ions into the SnSe and STO layers, density-function theory (DFT) calculations were carried out. As shown in Figure 5a,b, SnSe provided a faster diffusion channel for both the Cu and Ag ions as the energy barriers were 0.46 eV for the Cu ions and 0.38 eV for the Ag ions, respectively, which were much lower compared to that in STO. The energy barrier for the Ag ion diffusion in STO layer was 1.21 eV, which is relatively higher than that of the energy barrier for Cu ion, which was 0.99 eV, as shown in Figure 5c,d. This result supports the fact that Ag, as the top electrode, can archive more resistance states in the SnSe/STO heterostructure because the relatively higher energy barrier in STO helps to stabilize the intermediate states by delaying the filament rapture process. Since the LRS of devices is the initial state to achieve multistate and the time it takes for a voltage pulse to reach the middle state is approximately 1–5 µs [23], the filament in the SnSe layer and STO layer cannot migrate back to the top electrode completely in such a short time [22,47,48,49,50]. The filament rapture process continues at the SnSe/STO interface under increased negative voltage pulses on the top electrode, leading to a further change in the resistance state of the device. Therefore, this delayed process increases the possibility of forming more resistance states when negative voltage pluses are applied on the device to rupture the filament [13]. Figure 5e depicts the potential switching mechanisms of the formation and rupture of the conductive filament for the Cu and Ag electrode devices [51,52]. These intermediate states demonstrate that the devices are both resistive switching memories and memristors.

**Figure 6:**
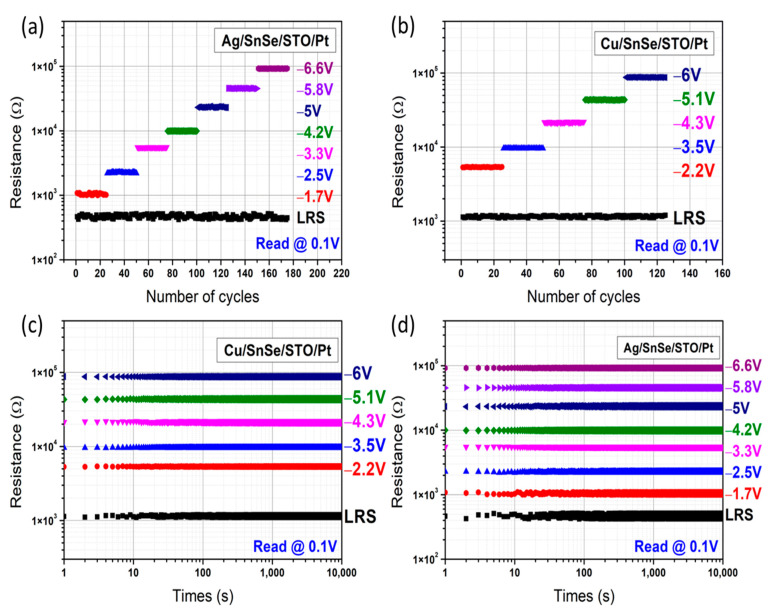
(**a**) The endurance characteristics of LRS and multilevel HRS under different RESET negative voltage pulses of the Cu/SnSe/STO/Pt device and (**b**) Ag/SnSe/STO/Pt device for 25 cycles each; (**c**) multilevel retention characteristics of LRS and multilevel HRS under different RESET negative voltage pulses of the Cu/SnSe/STO/Pt device and (**d**) Ag/SnSe/STO/Pt device for 10^4^ s.

Multilevel resistance states depending on different SET and RESET processes were investigated to find out the influence of positive and negative voltage pulses on the filament evolution in the resistive layers. As shown in Figure 6a, the LRS of the Cu/SnSe/STO/Pt device was achieved by positive voltage pulses, while the other HRS were achieved by negative voltage pulses. In the multistate test of the Cu/SnSe/STO/Pt device, a positive sweeping voltage from 0 to 2 V without current compliance was first applied to reach the LRS (state ‘5’), then a negative voltage pulse of −2.2V was applied to reach the first HRS (state ‘4’). With the gradual increase in negative voltage pulses to −6 V, the Cu/SnSe/STO/Pt device showed up to six resistance states. We also carried out a similar test to investigate the multistate characteristics of the Ag/SnSe/STO/Pt device and the results are shown in Figure 6b. One can see that the Ag/SnSe/STO/Pt device also switched from the initial state to LRS (state ‘7’) by positive voltage sweeping from 0 to 2 V, but its first HRS (state ‘6’) was achieved by a negative voltage pulse of −1.7 V, which was slightly lower than the Cu/SnSe/STO/Pt device. With the gradual increase in negative voltage pulses to −6.6 V, the Ag/SnSe/STO/Pt device presented up to eight resistance states (i.e., two more states than the Cu/SnSe/STO/Pt device). Normally, a device with more resistance states should perform better when applied in neuromorphic computing.

The stability of different resistance states was confirmed by the endurance test for 25 cycles of positive voltage sweeping and the negative voltage pulses to SET and RESET the devices, as shown in Figure 6a. For the Cu/SnSe/STO/Pt device, five high resistance states were found to be 1.12, 5.33, 9.65, 21.54, 43.37, and 87.72 kΩ at the RESET voltage pulse when sweeping from −2.2 V to −6 V. For the Ag/SnSe/STO/Pt device shown in Figure 6b, there were seven high resistance states from 1.05, 2.26, 5.53, 9.92, 23.55, 45.72, to 92.63 kΩ at RESET voltage pulses from −1.7 to −6.6 V.

The retention properties of the multistate memory cells for both devices were also investigated for the different resistance states, which were set by the former processes. As shown in Figure 6c,d, for both devices read at 0.1 V, all of the resistance states could last for 10^4^ s without an obvious change from the beginning to the end of the test. From these endurance and retention tests, we can conclude that the Cu/SnSe/STO/Pt and Ag/SnSe/STO/Pt memory devices are suitable for high-density storage and multilevel RRAM and memristor applications.

## 4. Conclusions

In summary, we fabricated and characterized SnSe/STO heterostructure-based memristors with different top electrodes. It was found that the resistance states of memory cells with a Cu top electrode and Ag top electrode could achieve six and eight resistance states, respectively, and the formation and destruction of Cu and Ag filaments in the SnSe and STO layers under voltage sweeping and voltage pulses are believed to be responsible for these characteristics. These multilevel states were reliable during 10^3^ SET and RESET reliability tests and were stable after 10^4^ s retention tests. These results suggest that the SnSe/STO heterostructure-based memristors may have a great potential for synaptic devices.

## Figures and Tables

**Table 1:** A comparison of the devices with the polycrystalline/crystalline STO film.

Device	Crystalline STO Film	Top Electrode	SET Voltage	RESET Voltage	Resistance Ratio	Resistance State	Ref.
Fe:STO/Nb:STO	Yes	Pt	~2.5 V	~−2.5 V	60	2	[18]
STO/SRO	Yes	Pt	~2 V	~−2 V	~100	2	[32]
STO/SRO	Yes	-	>−4 V	>−4 V	~10,000	2	[33]
Ba:STO/SRO	Yes	Pt	>−3 V	>4 V	~3	2	[34]
STO/Pt	Yes	Ag	~1 V	~−3 V	~100,000	4	[23]
SnSe/STO/Pt	Yes	Ag	<1 V	<−1 V	~250	8	*
SnSe/STO/Pt	Yes	Cu	~1 V	~−1 V	~100	6	*

* This work.
